# Profiling of Genes Related to Cross Protection and Competition for *NbTOM1* by HLSV and TMV

**DOI:** 10.1371/journal.pone.0073725

**Published:** 2013-09-04

**Authors:** Yi Wen, Grace Xiao-Yun Lim, Sek-Man Wong

**Affiliations:** 1 Department of Biological Sciences, National University of Singapore, Singapore, Singapore; 2 Temasek Life Sciences Laboratory, 1 Research Link, Singapore, Singapore; 3 National University of Singapore Suzhou Research Institute, Suzhou, Jiangsu, China; Nanyang Technological University, Singapore

## Abstract

Cross protection is the phenomenon through which a mild strain virus suppresses symptoms induced by a closely related severe strain virus in infected plants. Hibiscus latent Singapore virus (HLSV) and Tobacco mosaic virus (TMV) are species within the genus tobamovirus. HLSV can protect *Nicotiana* *benthamiana* against TMV-U1 strain, resulting in mild symptoms instead of severe systemic necrosis. The mechanism of cross protection between HLSV and TMV is unknown. In the past, some researchers suggest that the protecting virus strain might occupy virus-specific replication sites within a cell leaving no room for the challenge virus. Quantitative real-time RT-PCR was performed to detect viral RNA levels during cross protection. HLSV accumulation increased in cross protected plants compared with that of single HLSV infected plants, while TMV decreased in cross protected plants. This suggests that there is a competition for host factors between HLSV and TMV for replication. To investigate the mechanism under the cross protection between HLSV and TMV, microarray analysis was conducted to examine the transcriptional levels of global host genes during cross protection, using Tobacco Gene Expression Microarray, 4x44 k slides. The transcriptional level of some host genes corresponded to accumulation level of TMV. Some host genes were up-regulated only by HLSV. Tobamovirus multiplication gene 1 (*TOM1*), essential for tobamovirus multiplication, was involved in competition for replication by HLSV and TMV during cross protection. Both HLSV and TMV accumulation decreased when *NbTOM1* was silenced. A large quantity of HLSV resulted in decreased TMV accumulation in HLSV+TMV (100:1) co-infection. These results indicate that host genes involved in the plant defense response and virus multiplication are up-regulated by challenge virus TMV but not by protecting virus HLSV during cross protection.

## Introduction

Cross protection is a phenomenon through which a mild strain virus suppresses symptoms induced by a closely related severe strain virus in infected plants [1–4]. Earlier studies reviewed the mechanism of cross protection between the wild type strain and its mutants or different strains from the same virus strain [1,5–9]. The mechanisms of cross protection among strains of the same virus have been well described as coat protein (CP)-mediated resistance, replicase-mediated cross protection or RNA silencing [2,10–12]. Cross protection has been studied using two different tobamoviruses, instead of two strains. Wild type Sunn-hemp mosaic virus (SHMV) could protect host against a SHMV mutant encapsidated with Tobacco mosaic virus C (TMV-C) CP and also provided weak protection against TMV-C [13]. By *in vitro* study, it was shown that Brome mosaic virus (BMV) CP could encapsidate TMV RNA and interfere with virus replication by obscuring its replication recognition site [14].

In early cross protection studies, some researchers suggested that protective viruses might occupy virus-specific replication sites within a cell leaving no room for the challenge virus [15–21]. However, there has been no evidence to support this hypothesis. Host proteins are essential for plant virus multiplication. Tobamovirus multiplication gene 1 in *Arabidopsis thaliana* (*AtTOM1*), a seven-pass membrane protein, interacts with replication protein encoded by TMV-Cg [22]. There are several homologues of *AtTOM1* involved in tobamovirus replication [23–25]. ADP-ribosylation factor-like 5B in *Nicotiana* *tobacum* (*NtARL8*), a small host GTP-binding protein, is also required for tobamovirus multiplication [26].

Tobamovirus has one genomic RNA (gRNA) and three sub-genomic RNAs, sgRNA1 sgRNA2 and sg-coat protein RNA (LMC) [27]. However, only sgRNA2 and LMC are active as mRNAs in plants [28], from which six open reading frames (ORFs) are processed [29]. Hibiscus latent Singapore virus (HLSV) [30] and TMV are species within the genus tobamovirus. HLSV can infect *Hibiscus* and *Nicotiana* *benthamiana* plants. TMV can infect different tobacco species and induce different symptoms. Specifically, TMV induces systemic necrosis in *N* *. benthamiana* . Since HLSV causes mild mosaic symptoms, we tested whether HLSV can cross protect *N* *. benthamiana* against TMV and to investigate the mechanism. We also examined if there is a competition between HLSV and TMV for *NbTOM1*which is essential for tobamovirus replication.

Microarray technology was used for global analysis of gene transcriptional levels among inoculation buffer (mock)-, HLSV-, TMV- and HLSV+TMV-inoculated *N* *. benthamiana* . To date, up to 44,000 genes and expressed sequence tag (EST) can be monitored in parallel. As *N* *. benthamiana* gene expression microarray chips were not available, we had to compromise and used the closest tobacco gene expression microarray chips for *N. tabacum* genes, 4x44 k chip, to determine the transcriptional level of host gene changes in *N* *. benthamiana* before and after TMV infection, while plants were pre-inoculated with HLSV.

## Results

### Symptoms of HLSV and TMV infection in *N. benthamiana*

**Figure 1:**
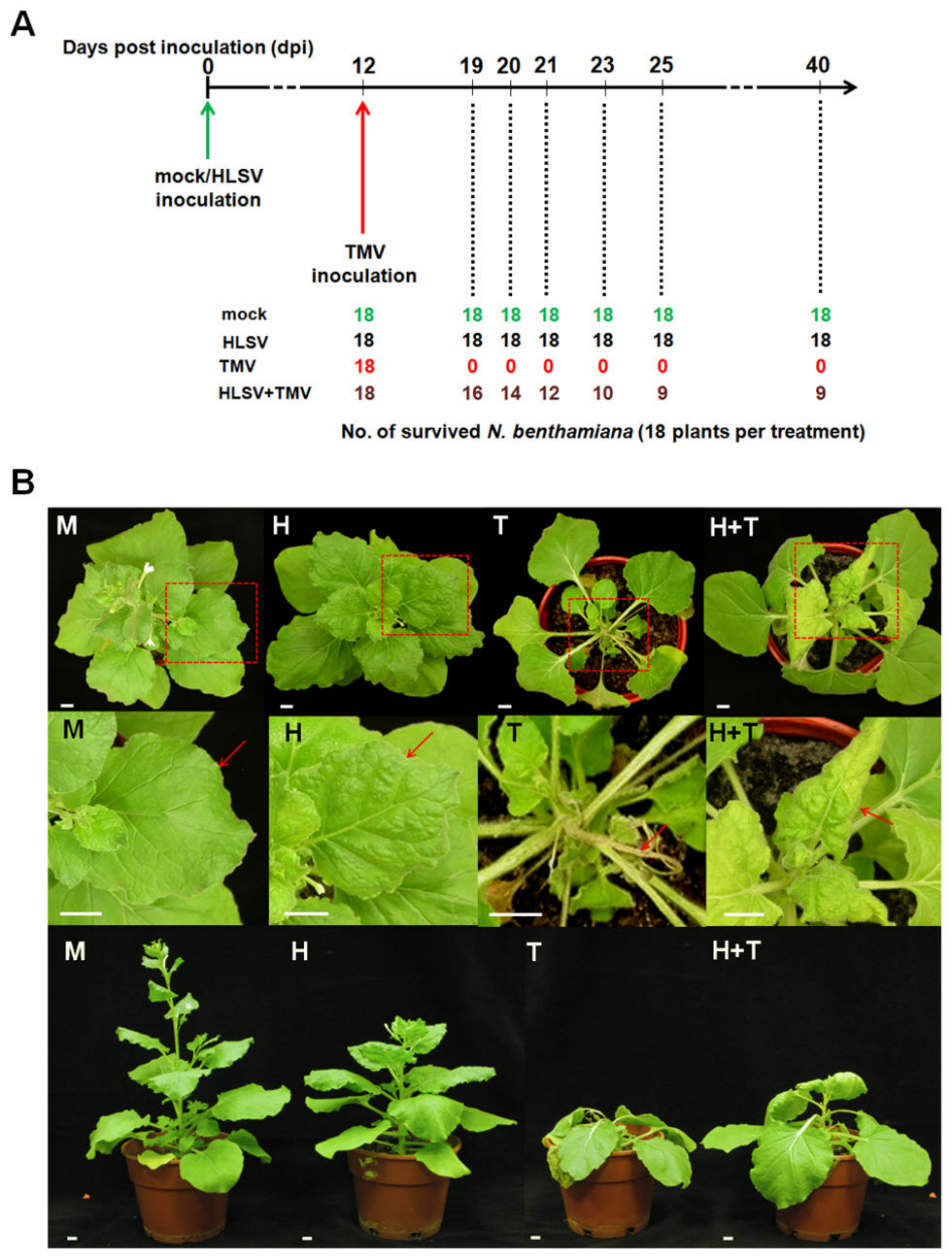
Cross protection between HLSV and TMV and its symptom expressions in *Nicotiana* *benthamiana* . (A) The number of surviving *N* *. benthamiana* plants inoculated with mock buffer, HLSV, TMV and HLSV+TMV at different time points. All plants survived mock and HLSV inoculation. No plants survived inoculation with TMV. Half of the TMV infected *N* *. benthamiana* (pre-inoculated with HLSV 12 days earlier) survived at 40 dpi. (B) The top, middle and bottom rows showed the top, close-ups and the side views of mock buffer (M), HLSV (H), TMV (T) and HLSV+TMV (H+T) inoculated *N* *. benthamiana* plants, respectively. The red dotted line boxes in the top panels highlighted the close-up areas shown in the middle panels. Typical symptoms (red arrows pointing to) of inoculated *N* *. benthamiana* plants are shown in the middle row. Among them, panel M, no symptom; panel H, mild leaf puckering at 20 dpi; panel T, systemic necrosis (plant death) at 8 dpi; panel H+T, mild mosaic symptoms at 20 dpi, plant height was shorter than H but taller than T inoculated plants. All scale bars represent 1 cm.

Cross protection between HLSV and TMV was examined in *N* *. benthamiana* . TMV caused severe cell death on upper newly expanded leaves one week post inoculation, which was reduced and delayed by pre-inoculation with HLSV. At the same time, mock buffer or HLSV inoculation did not cause any cell death in *N* *. benthamiana* (Figure 1A). HLSV caused mild leaf puckering symptoms at 12 days post-inoculation (dpi) and similar symptoms were observed at 20 dpi [Figure 1B (H), top and middle panels]. TMV caused systemic necrosis in upper newly expanded leaves and stems of infected plants at 8 dpi [Figure 1B (T), top, middle and bottom panels]. TMV was inoculated into *N* *. benthamiana* leaves which were inoculated with HLSV 12 days earlier. The systemic necrosis was delayed; light green mosaic and curling symptoms were observed on the upper newly expanded leaves at 8 days post TMV infection [Figure 1B (H+T), top and middle panels]. The growth of *N* *. benthamiana* was retarded by HLSV infection [Figure 1B (H), bottom panel], as compared with that of mock buffer inoculated [Figure 1B (M), bottom panel]. TMV reduced plant growth dramatically [Figure 1B (T), bottom panel]. The height of cross protected plants was greater than the TMV infected plants but shorter than that of HLSV infected plants [Figure 1B (H+T), bottom panel].

### Detection of HLSV and TMV RNA levels by quantitative real-time reverse transcription (RT) PCR and coat proteins by western blot during cross protection in *N. benthamiana*

**Figure 2:**
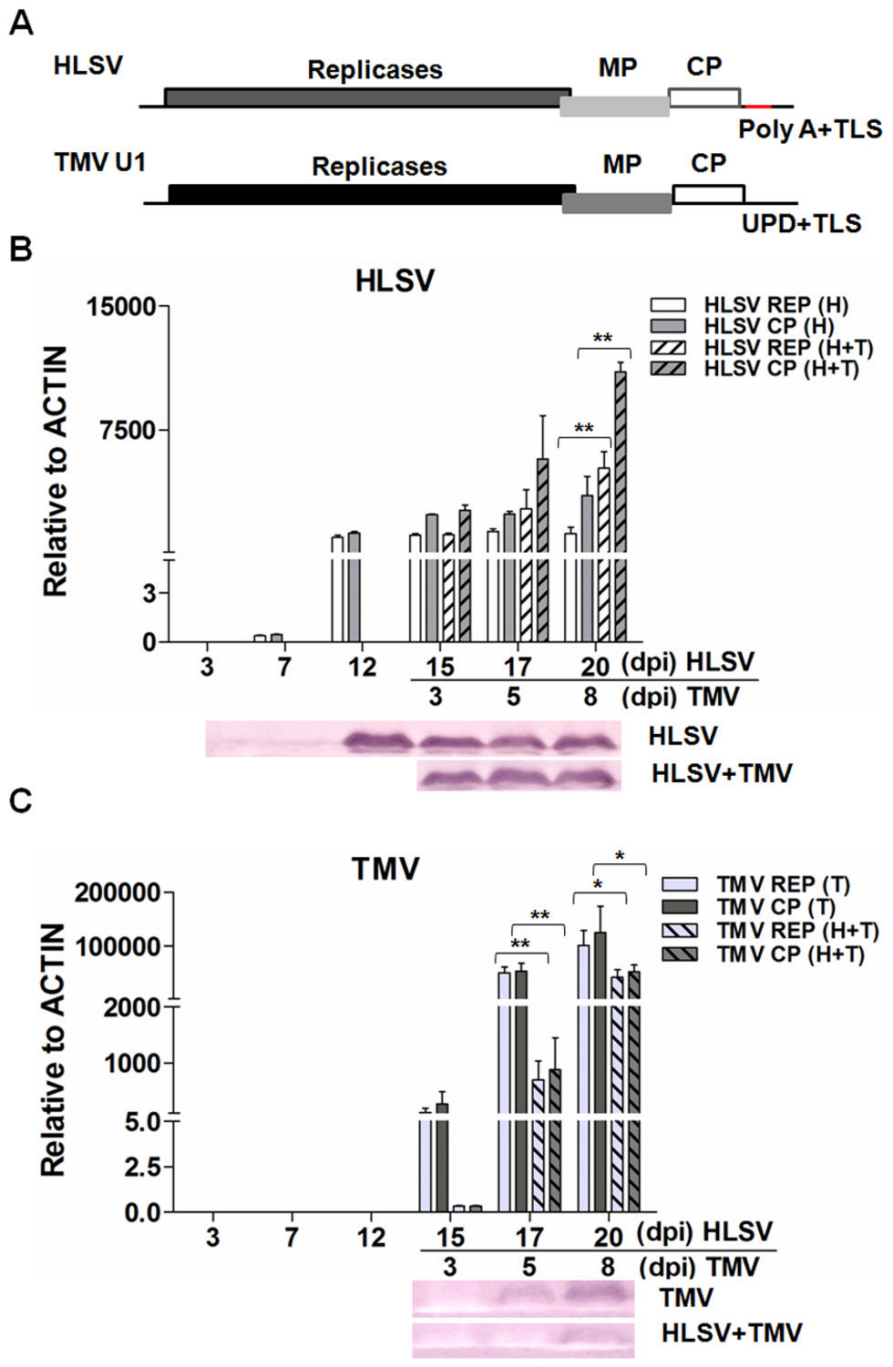
Genome organizations of HLSV and TMV and detection of viral RNA and protein levels in cross protection. (**A**) Genome organization of HLSV and TMV. Transcriptional level of HLSV (**B**) or TMV (**C**) gRNA/total viral RNA determined by quantitative real-time RT-PCR and translational level of CPs by western blot (**B** and **C**). Significant differences were calculated using the Student’s *t*-test, * and ** indicate significance at the 0.05 and 0.01 levels of confidence, respectively.

HLSV processes a poly(A) tract instead of an upstream pseudoknot domain (UPD) in the upstream of a t-RNA like structure (TLS) in the 3’-end of HLSV RNA [30]. The PCR products amplified from specific primers responding to the replication protein genes represent the total amount of viral genomic RNAs (gRNAs), whereas the PCR products amplified from primers corresponding to the CP genes represent the amount of total viral RNAs of HLSV and TMV (Figure 2A), respectively. The detailed information of primers for quantitative real time RT-PCR is listed in Table S1. The transcript levels of HLSV replication protein gene and CP gene increased in HLSV protected *N* *. benthamiana* plants (H+T) compared with those infected with HLSV alone (H) (Figure 2B upper panel). At the same time, the transcript level of TMV decreased compared with those of infected by TMV alone (T) (Figure 2C upper panel). The CP detected by western blot corresponded with the results of viral RNA levels (Figure 2B & 2C, lower panels). Significant differences were calculated using the Student’s *t*-test, * and ** indicate significance at the 0.05 and 0.01 levels of confidence, respectively.

### Microarray results

**Figure 3:**
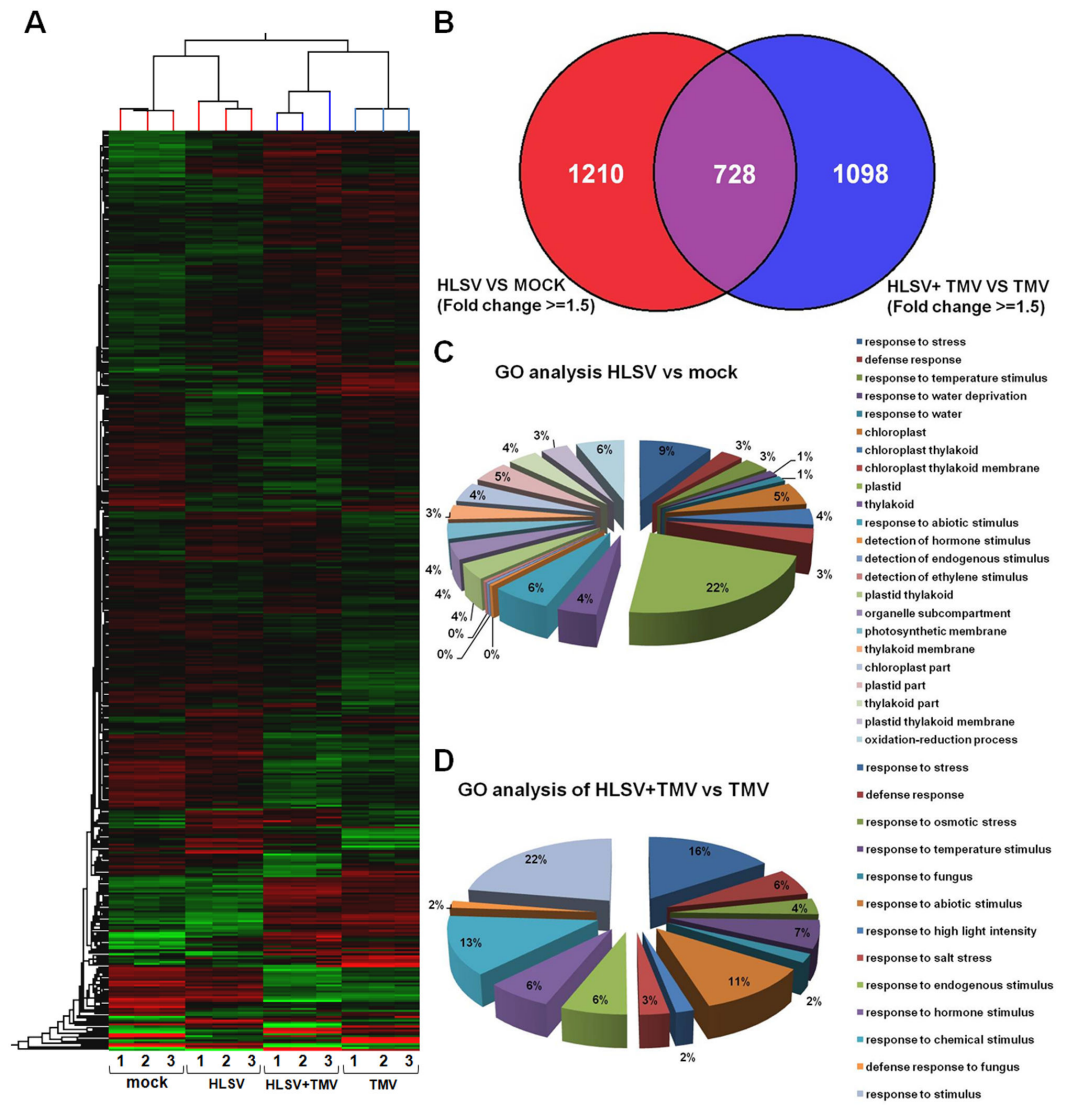
Hierarchical cluster analysis of transcriptional profiles and gene ontology analysis. (A) A hierarchical cluster analysis of transcriptional profiles for 24237 genes or ESTs of *Nicotiana* *benthamiana* inoculated with inoculation buffer (mock), HLSV, HLSV+TMV (plants cross protected by pre-inoculation of HLSV 12 days prior to TMV challenge inoculation). The 3 columns correspond to biological repeats after inoculation with buffer (mock), HLSV, HLSV+TMV and TMV (at 12 dpi for mock and HLSV, and 3 dpi for HLSV+TMV and TMV which is equal to 15 dpi of HLSV). The clustering on the top of hierarchical map represents the differences among samples and biological repeats, while the left clustering is based on the expression levels of different genes. (B) Venn diagram of genes in response to HLSV infection and cross protection. (C) HLSV infection. (D) Gene ontology (GO) analysis of genes in response to HLSV+TMV infection (cross protection).

For an overview of global changes in transcriptional level of host genes before and after cross protection between HLSV and TMV, a microarray analysis was performed using tobacco (*N. tabacum*) chips (Agilent Technologies, USA). Hierarchical cluster analysis of transcriptional profiles for 24,237 genes or expressed sequence tags (ESTs) for *N* *. benthamiana* inoculated with buffer (mock), HLSV, TMV and HLSV+TMV (cross protected plants pre-inoculated with HLSV 12 days earlier) was performed. Gene expression profiles for each of the 3 individual biological repeats (1–3) after inoculation with buffer (mock), HLSV, TMV and HLSV+TMV are presented in Figure 3A. Mock and HLSV samples were taken at 12 dpi. HLSV+TMV and TMV samples were taken at 3 dpi by TMV (15 days post inoculation with mock or HLSV). At the transcriptional level 1,938 genes were changed in response to HLSV infection and 1,826 genes were changed in response to cross protection by HLSV, as shown in the Venn diagram. For group HLSV vs mock and group HLSV+TMV vs TMV, 728 genes were overlapped. The gene ontology (GO) analysis of genes in response to HLSV infection and cross protection was obtained (Figure 3C and 3D). Compared with the mock inoculated *N* *. benthamiana* , genes changes were noted at the transcriptional level in HLSV infected plants (corrected P value < 0.01), including those genes that respond to stress, defense, temperature stimulus, water deprivation, abiotic stimulus, detection of hormone, endogenous and ethylene stimuli, oxidation-reduction process or function in chloroplast, thylakoid, thylakoid membrane, plastid, and organelle sub-compartment (Figure 3C). Moreover, there were changes in transcriptional level of host genes in cross protected *N* *. benthamiana* (corrected P value < 0.01) compared with TMV infection. These genes responded to stress, defense, osmotic stress, temperature stimulus, fungus, abiotic stimulus, high light intensity, salt stress, endogenous/hormonal and chemical stimuli. The microarray data has been submitted to database GEO under the NCBI database and an accession number GSE47180 is assigned.

### Validation of selected microarray data

Due to the large number of probes on each tobacco microarray chip, it would not be feasible to verify all of them by quantitative real-time RT-PCR. Hence, a subset of genes was selected for validation. To validate the microarray data and to understand the mechanism of cross protection between HLSV and TMV, a general approach was adopted to focus on genes that either showed large-fold change values between HLSV+TMV and TMV samples involved in general plant defense pathways at 15 dpi of HLSV [31–39], or host factors that have been implicated in interactions with tobamoviruses [22–24,40–48].

A standard curve of primer dilutions against C_T_ values was plotted to calculate the amplification efficiencies of primers. All amplifications efficiencies of the primers used were determined to range from 90% to 110% (Figure S1).

Argonaute 4-2 (*NbAGO4-2*), auxin repressed protein 1 (*NbARP1*), calmodulin 3 (*NbCaM3*), cysteine protease 2 (*NbCP2*), proteinase inhibitor (NbPI), 1-aminocyclopropane-1-carboxylate oxidase (*NbACO*), *NbALY*, CCR4-associated factor 1 (*NbCAF1*), catalase 1 (*NbCAT1*), heat shock protein 101 (*NbHSP101*), systemic acquired resistance 8.2m (*NbSAR8.2m*), wound induced protein kinase (NbWIPK), double WRKY type transfactor protein (*NbWRKY7*and *NbWRKY8*), *NbTOM1*, vacuolar processing enzyme 1 α (*NbVPE1α*) were validated using quantitative real-time RT-PCR. The magnitude of changes of selected genes obtained by quantitative real-time RT-PCR was in agreement with the values obtained from the microarray data (Table 1).

**Table 1:** Validation of microarray data in fold changes using quantitative real-time RT-PCR.

	**HLSV vs Mock (12 dpi)**		**HLSV+TMVvs TMV(15 dpi)**		
**Gene**	**Real time RT-PCR**	**Microarray**	**Real time RT-PCR**	**Microarray**	**Validated**
*NbAGO4-2*	1.29	1.04	1.23 to 1.51	-1.06	Yes
*NbARP1*	1.03	-1.17 to 1.14	2.51	1.84 to 2.06	Yes
*NbCaM3*	1.49	2.33 to 2.97	2.06	2.54 to 4.57	Yes
*NbCP2*	3.15	2.78 to 3.60	6.66	3.20 to 4.67	Yes
*NbPI*	1.64	2.26 to 2.31	-3.19	-9.14 to -9.29	Yes
*NbACO*	1.63	1.95	-1.52	-16.17	Yes
*NbALY*	1.26	-1.06 to 1.01	-1.19 to 1.17	1.06 to 1.13	Yes
*NbCAF1*	-1.50 to 1.64	-1.05 to 12.61	-1.21	-1.02 to 11.35	Yes
*NbCAT1*	1.65	2.19	1.95	3.01	Yes
*NbHSP101*	1.35	-2.34	-1.33	-17.14	Yes
*NbSAR8.2m*	1.21	1.72 to 1.80	-18.00	-6.90 to -6.86	Yes
*NbWIPK*	1.66	-1.70	-1.97	-3.62	Yes
*NbWRKY7*	-1.39	-1.09	-5.89	-1.06	Yes
*NbWRKY8*	-1.23	-1.74	-2.76	-3.55	Yes
*NbTOM1*	-1.05 to 1.65	1.29	1.32	1.40	Yes
*NbVPE1α*	2.31	2.39	1.07 to 2.28	1.84	Yes

### Transcriptional levels of selected genes during cross protection

**Figure 4:**
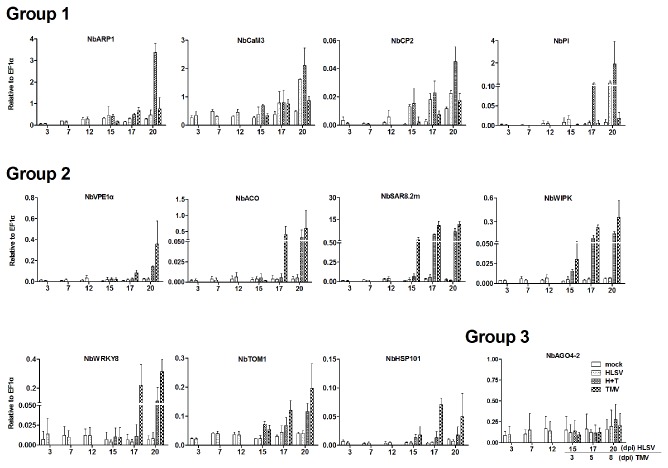
Transcriptional levels of selected genes in cross protection. The transcriptional levels of *NbARP1*, *NbCaM3*, *NbCP2*, *NbPI*, *NbVPE1α*, *NbACO*, *NbSAR8.2m*, *NbWIPK*, *NbWRKY8*, *NbTOM1, NbHsp101* and *NbAGO4-2*.

A 20-day time course experiment was conducted to monitor the transcriptional level of selective genes, including *NbARP1*, *NbCaM3*, *NbCP2*, *NbPI*, *NbVPE1a, NbACO*, *NbSAR8.2m*, *NbWIPK, NbWRKY8*, *NbTOM1, NbHsp101* and *NbAGO4-2* (Figure 4). The transcriptional level of host genes could be classified into three groups. In Group 1, the transcriptional levels of different genes were elevated in cross protected plants (HLSV+TMV) and/or HLSV infected plants. These genes included *NbARP1*, *NbCaM3*, *NbCP2* and *NbPI*. In group 2, the transcriptional level of different host genes (*NbVPE1a, NbACO*, *NbSAR8.2m*, *NbWIPK, NbWRKY8*, *NbTOM1* and *NbHsp101*) increased in single TMV infected plants rather than those of mock inoculated, HLSV infected or cross protected plants (HLSV+TMV). Additionally, the transcriptional level of the gene (*NbAGO4-2*) in Group 3 was unchanged in upper newly expanding leaves either from mock inoculation buffer, HLSV, TMV, or HLSV+TMV inoculated plants.

### Competition of *NbTOM1* between HLSV and TMV

**Figure 5:**
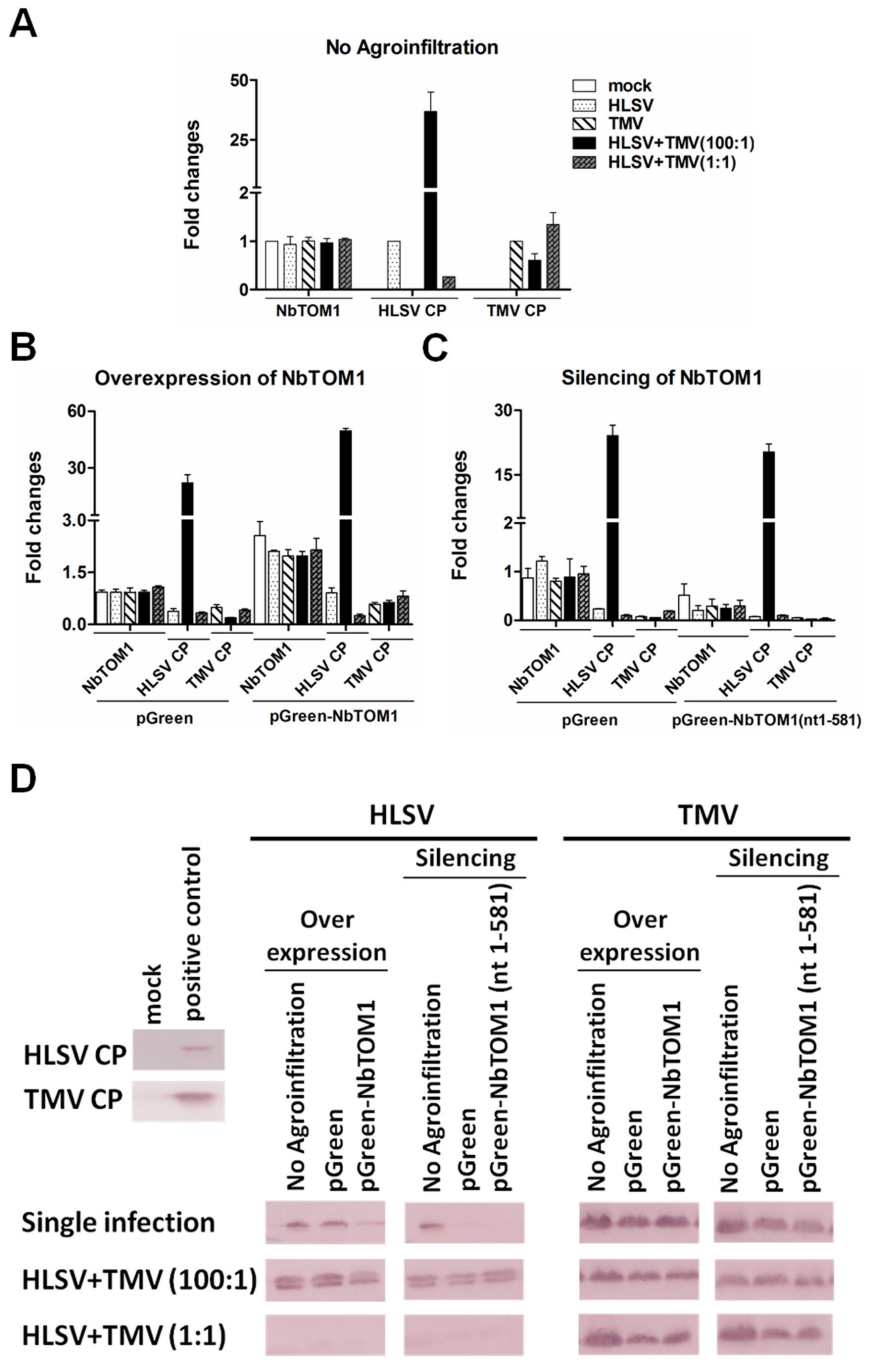
*NbTOM1* transcript levels and virus accumulation with overexpression or silencing of *NbTOM1* in *Nicotiana* *benthamiana* . (A) The transcriptional levels of *NbTOM1* were detected in mock inoculation buffer, HLSV, TMV, HLSV+TMV (100:1) and HLSV+TMV (1:1) co-infected plants. The viral RNA levels of HLSV and TMV were determined using quantitative real-time RT-PCR with primer sequences corresponding to the coat protein genes in HLSV, or TMV or co-infected leaves. (B and C) The transcriptional levels of *NbTOM1* were detected in *NbTOM1*overexpressed or silenced leaves. The viral RNA levels were detected in plants first infiltrated with pGreen orpGreen-*NbTOM1* (for overexpression), and pGreen or pGreen-*NbTOM1*(nt1-581) (for silencing), followed by single virus (HLSV or TMV) infection or co-infection(HLSV+TMV) at 40 h post inoculation (hpi). (D) The coat proteins of HLSV and TMV were detected by western blot in *NbTOM1*overexpressed or silenced leaves which were subsequently infected with single virus (HLSV or TMV) or co-infected with HLSV+TMV at 5 dpi (details see Materials & Methods). Total protein from mock buffer inoculated *N* *. benthamiana* leaves was used as the negative control, while the total protein from HLSV or TMV infected leave samples which were confirmed earlier were used as positive controls.

To test if *NbTOM1* was essential for HLSV infection and if it was competed by HLSV and TMV, inoculation buffer (mock), HLSV, TMV, HLSV+TMV (100:1) and HLSV+TMV (1:1) were inoculated into *N* *. benthamiana* either with no Agro-infiltration, overexpression of *NbTOM1* or silencing of *NbTOM1*. At 40 h post inoculation (hpi), the transcriptional levels of *NbTOM1* were found to be similar among the different treatments (Figure 5A). There was up-regulation (2X) of *NbTOM1* in pGreen-*NbTOM1* infiltrated leaves (Figure 5B). Additionally, the transcriptional level of *NbTOM1* decreased in *NbTOM1*-silenced leaves which were Agro-infiltrated with pGreen-*NbTOM1*(nt1-581) (Figure 5C). The transcriptional level of *NbTOM1* in buffer inoculated leaves (mock) without Agro-infiltration was set as a baseline (to the value of 1) for the transcriptional level of *NbTOM1*. In Agro-infiltrated leaves with pGreen alone, the transcriptional level of *NbTOM1* was similar to that of mock inoculated leaves. This indicated that the transcriptional level of *NbTOM1* did not change with Agro-infiltration of the empty vector alone.

The RNA level of HLSV and TMV in non-Agro-infiltrated leaves was also set as a baseline individually (to the value of 1) for comparison with HLSV alone, TMV alone, and HLSV+TMV co-infection with overexpression and silencing of *NbTOM1*. In non-Agro-infiltrated plants inoculated with HLSV+TMV (100:1), the TMV RNA level decreased, as compared with that of single TMV infection. At the same time, in HLSV+TMV (1:1) co-infected leaves, the TMV accumulation increased (Figure 5A). These results indicated that the amount of HLSV affected TMV accumulation. Additionally, the accumulation level of HLSV decreased in HLSV+TMV (1:1) co-infection (Figure 5A), which indicated that competition occurs in HLSV+TMV co-infection.

In the leaves Agro-infiltrated with the pGreen empty vector alone, virus accumulation decreased, as compared with non-Agro-infiltrated *N* *. benthamiana* (Figure 5B and 5C). With the overexpression of *NbTOM1*, HLSV accumulation increased in single infection and HLSV+TMV (100:1) co-infection but decreased in HLSV+TMV (1:1) co-infection (Figure 5B). At the same time, TMV amount also increased slightly in two co-infections, when compared with that of leaves infiltrated with pGreen vector alone. Moreover, HLSV and TMV accumulation decreased with silencing of *NbTOM1* (Figure 5C). These results indicated that *NbTOM1* was essential for HLSV and TMV replication and its up-regulation (2X) favored both virus replications (Figure 5B).

The CP levels of two viruses were determined using HLSV or TMV CP antibody. The TMV CP level decreased in HLSV+TMV (100:1), as compared with that of single TMV infected leaves at 5 dpi. HLSV CP level decreased in HLSV+TMV (1:1) infected leaves. This indicated that only large amount of HLSV inocula or higher level of its accumulation allowed it to compete effectively for *NbTOM1* with TMV.

## Discussion

HLSV and TMV-U1 are species of genus tobamovirus. HLSV causes mild puckering and slight mosaic symptoms in *N* *. benthamiana* , while TMV causes severe systemic necrosis. In our study, we found that HLSV can cross protect *N* *. benthamiana* against TMV, resulting in milder symptoms. Quantitative real-time RT-PCR and western blot were performed to detect the level of genomic RNA alone and total viral RNAs and CPs during cross protection. HLSV accumulation increased in cross protected plants, as compared with that of single HLSV infected plants, while TMV accumulation decreased in cross protected plants. These results suggested that in cross protected plants there might be changes in the transcriptional level of some host genes that can enhance HLSV accumulation. Moreover, the results also suggest that pre-infection of HLSV allowed it to compete with TMV more favorably by early sequestering of host factors for replication.

To understand the transcriptional changes in cross protection, we conducted a microarray analysis using tobacco chips. With HLSV infection in *N* *. benthamiana* , some genes changed in transcriptional levels which responded to stress, defense, environmental and hormone stimuli, oxidation-reduction process or function in organelles. Furthermore, the transcriptional levels of host genes responded to stress, defense, osmotic stress, environmental, hormone, biotic and abiotic stimuli at the beginning of cross protection. We further investigated the transcriptional patterns of selective host genes.

In Group 1, host genes *NbARP1*, *NbCaM3*, *NbCP2* and *NbPI* were up-regulated in cross protected or HLSV-infected *N* *. benthamiana* . Auxin-response genes are controlled by auxin, resulting in regulating various growth and development [49]. To date, no information is available on *ARP1* related to plant defense. The CaM3 gene in *N. tabacum* activates NAD kinase which is also a cofactor of NADPH oxidase in producing reactive oxygen species (ROS) [50]. In view of the higher levels transcription of *NbCaM3* in cross protected *N* *. benthamiana* , as compared to TMV-infected plants, this suggests that ROS signaling is activated during cross protection. High level of ROS induced oxidation to carbohydrates, lipids, proteins, and DNA. ROS also influence a few plant hormone responses and affect many processes, such as systemic signaling, growth, development, abiotic stress responses, pathogen defense and programmed cell death (PCD) [51]. The *CP2* belongs to the C1 family of papain-like cysteine proteases and in *N. tabacum*, it shares high amino acid similarity (68–72%) with KDEL-tailed plant cysteine proteases, which may be involved in PCD [52]. In addition, the KDEL motif may enhance vacuolar transport. The role of *CP2* in TMV infection has not been elucidated. Interestingly, the transcriptional level of *NbCP2* was correlated with HLSV infection and was much higher in cross protected plants than single TMV infected *N* *. benthamiana* . Proteinase inhibitor (NbPI) was also up-regulated in cross protected *N* *. benthamiana* . Plant cysteine proteases play an instrumental role in PCD and *PI* may prevent unwanted cell death [53]. In cross protected *N* *. benthamiana* , the up-regulation of *NbPI* may function in preventing necrosis caused by TMV infection. Further studies can be conducted to ascertain the roles of *NbCP*2 and *NbPI* in cross protection.

Moreover, host genes (*NbVPE1α, NbACO*, *NbSAR8.2m*, *NbWIPK*, *NbWRKY8*, *NbTOM1* and *NbHSP101*) in Group 2 were up-regulated in TMV infected *N* *. benthamiana* . The necrotic lesions at the sites of TMV infection is considered PCD in tobacco plants with *N* gene [54]. *VPE1α* belongs to the caspase-like proteases. During TMV infection, *VPE1α* exhibits caspase-1 activity in TMV-infected tobacco leaves. Cell death was abolished upon treatment with caspase-1 and VPE inhibitors. Increased virus accumulation was also observed in RNAi VPE-silenced *N* *. benthamiana* with temperature shift [45]. In cross protected *N* *. benthamiana* , the transcriptional level of *NbVPE1α* increased as compared with that of single HLSV infected *N* *. benthamiana* , but decreased as compared with that of plants infected with TMV alone. At present, it is unclear if the roles of *VPE1a* and *CP2* overlap in PCD.

The *NbACO* is involved in the final step of ethylene biosynthesis. Ethylene is substantially increased by infection with fungi in Citrus fruits, and this may be an active defense response. Up-regulation of ethylene biosynthesis genes can increase ethylene production [55]. The up-regulation of *NbACO* transcript level in TMV infected *N* *. benthamiana* indicates that ethylene production may also be increased in plants infected by TMV. The transcriptional level of *NbACO* is lower in cross protected or HLSV-infected *N* *. benthamiana* , as compared with that of plants infected with TMV alone, suggesting that ethylene production in this case is solely dependent on the accumulation level of TMV.

The hypersensitive response is accompanied by a marked increase in salicylic acid (SA) and rapid production of ROS [56]. Plants rely on circulating hormones to relay systemic signals when under pathogen attack [57]. SA is an important regulator in the plant defense system. Antiviral actions induced by SA include inhibitory effects on virus replication, cell-to-cell movement and long-distance movement [58]. Specific to TMV infection, SA interferes with replication of TMV at the point of inoculation and inhibits viral movement out of inoculated tissue [59]. Previously, pre-treatment of susceptible tobacco with SA reduced TMV accumulation significantly. To elucidate the role of SA in cross protection, three genes, *WIPK, WRKY8* and *SAR8.2m* were selected and monitored in buffer inoculated plants (mock), HLSV, HLSV+TMV (cross protected) and TMV infected plants. The transcriptional levels of these genes only dramatically up-regulated in plants infected solely with TMV and cross protected plants, but not in plants infected with HLSV alone. This indicates that the SA pathway is triggered whenever TMV is present.

Accumulation of the protecting virus may exclude the challenge virus from cells by occupying important sites or by depleting host factors required for replication of the challenge virus [2]. *TOM1* is a transmembrane protein in plants that serves as an attachment anchor for virus replication complexes in the host cell [22]. Subsequent studies [60] reaffirm it as an essential component of the tobamovirus replication complex, and silencing of the *TOM1* homologues gene results in reduced virus multiplication. HLSV may have a similar requirement for the *TOM1* protein; consequently, in cross protected plants TMV would have to compete with HLSV for *TOM1* and this could result in reduced accumulation of TMV. *TOM1* was up-regulated with TMV infection but not with HLSV infection. This may be due to lower level of HLSV replication which failed to up-regulate *TOM1*. Meanwhile, the up-regulation of *TOM1* in cross protected plants by TMV infection may facilitate HLSV replication, resulting in increased level of HLSV in cross protected plants. Heat shock protein 101 (*HSP101*) acts as a translational enhancer of TMV. It binds to the CAA-repeat motif of the 5’ Ω-region of the TMV RNA which recruits *eIF4* [61]. The CAA repeats are also found in the 5’-UTR of the HLSV RNA [30]. It is believed that HLSV can also interact with HSP101 in a similar manner as TMV with *HSP101*. Therefore, HLSV and TMV may also compete for *HSP101* in cross protected plants.

Although simultaneous RNA interference against *NtTOM1* and *NtTOM3* in *N. tabacum* resulted in nearly complete inhibition of the multiplication of tobamoviruses [25], we have chosen to focus on *NbTOM1*, a well studied host factor in tobamovirus replication, instead of *NtTOM3*, in *N* *. benthamiana* for investigation during cross protection. In the competition between HLSV and TMV for *NbTOM1*, TMV accumulation decreased in HLSV+TMV (with a ratio of 100:1) co-infected plants with and without overexpression of *NbTOM1* in comparison to TMV infection alone or HLSV+TMV (with a ratio of 1:1). This result confirms that there is competition for *NbTOM1* between HLSV and TMV for replication. Moreover, a larger amount of HLSV enhanced its competiveness with TMV (Figure 5). Increased amount of *NbTOM1* also led to increased virus accumulation of HLSV and TMV (Figure 5B). With silencing of *NbTOM1*, HLSV or TMV accumulation decreased either in both single infection and co-infection, which indicates that *NbTOM1* is essential for both HLSV and TMV accumulation. In conclusion, at low accumulation rate of HLSV, the plant defense response is triggered by the infection of TMV, but not by HLSV. Microarray validation showed that SA and ethylene pathways were enhanced after TMV infection. The host factor *NbTOM1* is important for tobamovirus multiplication, including HLSV. The changes of viral accumulation levels of HLSV and TMV in cross protected plants resulted from their competition for host factors notably including *NbTOM1*.

## Materials and Methods

### Plant materials and virus inoculation

*Nicotiana* *benthamiana* seeds were sown and seedlings were grown in a growth room with a 16 h photoperiod and 8 h darkness, at a constant temperature of 23°C. When the seedlings reached 6 to 8 fully expanded leaves, they were inoculated with mock inoculation buffer (0.01M phosphate buffer, pH 7.0) or HLSV by mechanical inoculation. Two leaves on each plant were dusted with carborundum and inoculated with mock buffer (negative control) or purified HLSV [62]. Similarly, two leaves on each plant were inoculated with TMV. For HLSV+TMV (cross protection), two leaves from each plant was first inoculated with HLSV 12 days before TMV inoculation. A total of 18 plants per treatment were used. For the detection of viral RNA/protein or transcriptional level of host genes, 6 plants per treatment were used. Three samples were taken from 3 plants from each treatment at different time points. HLSV or TMV (150 ng) were used for inoculation in cross protection experiments. For overexpression and silencing experiments, 100 times higher amount of HLSV was used in HLSV+TMV (100:1) co-infection. This was because the maximum amount of HLSV reached was approximately 1/10 of the maximum amount reached by TMV in *N. benthamiana*at 20 dpi (Figure 2B & C). Therefore, an elevated amount (100 times higher) of HLSV was used. Purified viruses of HLSV and TMV originated from their in vitro transcripts derived from biologically active full-length cDNA clones. The complete genome sequence of HLSV is deposited into the NCBI database (accession number NC_008310). TMV U1 strain (AF165190) was used for inoculation.

### RNA extraction and reverse transcription

Three youngest, HLSV systemically infected upper leaves (representing three individual biological repeats) were harvested for total RNA extraction at 3, 7, 12, 15, 17 and 20 days post inoculation (dpi) with inoculation buffer (mock), HLSV, TMV and HLSV+TMV (pre-inoculated with HLSV 12 days prior to inoculation of TMV). For overexpression and silencing of *NbTOM1*, three leaves from different plants first with Agro-infiltration, followed by virus inoculation, were harvested separately for RNA extraction, which represented three biological repeats. Leaf ( *N* *. benthamiana* ) samples were homogenized in liquid nitrogen, total RNA was extracted using the Trizol® Reagent (Invitrogen, USA). After the washing step, the total RNA pellet was air dried for 3 min and stored at -80°C. RNA was prepared using the same protocol for both microarray and quantitative real-time PCR experiments. Two µg of total RNA, oligodT_20_ and virus specific primers were used for first-strand cDNA synthesis using Superscript III® reverse transcriptase (Invitrogen, USA).

### Total protein extraction and western blot

Total proteins were extracted according to the same time points used for RNA extraction. Three leaves ( *N* *. benthamiana* ) were harvested and homogenized with one volume of protein extraction buffer (0.15M Tris-HCl (pH 6.8), 10% of glycerol, 5 mM DTT, 2% of SDS) and 1/5 volume of 5X SDS loading dye and denatured in water bath at 100 °C for 5 min. Then the total proteins were incubated on ice for 5 min and centrifuged at 16 x 1,000 *x g* at 4 °C for 5 min. In the *NbTOM1* competition experiment, total proteins were also extracted from Agro-infiltrated and virus infected leaves 5 dpi. The western blot was performed as previously described [63]. To avoid cross reactivity of antibodies of HLSV CP and TMV CP, 20,000 times dilutions of each antibody were used in western blots. At such a high dilution of antibody, there was no cross reactivity (unpublished data).

### Microarray hybridization and data analysis

Three biological repeats of RNA samples from the following four data points: mock and HLSV samples at 12 dpi; and HLSV+TMV (cross protected) samples and TMV samples at15 dpi with HLSV (equals 3 dpi of TMV) were prepared. These 12 samples were sent to Genomax Technologies Pte Ltd (Singapore) for microarray hybridization using Agilent’s Gene Expression, 4x44K format. The data analysis was carried out using GeneSpring GX 12.1. Gene Ontology (GO) analysis was carried out using Genespring 12.1 with GO annotation curated from consortium by Agilent Technologies (USA).

### Primer design and verification

Genes of interest with quantitatively significant changes in plant resistance and virus–host protein interactions were selected from the microarray results. First, the corresponding UniGene identifier for selected probes was entered into the database of NCBI, and *N. tabacum* gene sequence returned was queried using BLAST, to identify if there were partial or full length sequences of *N* *. benthamiana* homologues in the database of NCBI. If the desired sequences were available, software GenScript Real-time PCR Primer Design was used to design real-time primers. When identical genomic sequences in *N* *. benthamiana* were not available; primers based on conserved domains of known homologous sequences from other species were designed to obtain partial gene sequences of *N* *. benthamiana* . Sequence alignment was carried out using software SeqMan from Lasergene (http://www.dnastar.com). Partial sequences of *NbARP1* (KF051944), *NbCaM3* (KF051945), *NbCP2* (KF051946), *NbPI* (KF051947) and *NbHSP101* (KF051948) were obtained by the method described above. After these partial sequences were obtained through reverse transcription (RT)-PCR and sequenced, real-time primers were designed using the GenScript Real-time PCR Primer Design. All primers for quantitative real-time PCR were selected by considering the predicted RNA secondary structure from The Mfold Web Server (http://mfold.rna.albany.edu/?q=mfold/). Primers were synthesized by 1st Base Pte Ltd, Singapore. The desired gene fragments were amplified using first-strand cDNAs from healthy *N* *. benthamiana* as templates. Single band corresponding to the predicted fragment size was sequenced using Applied Biosystems 3130 Genetic Analyzer (Applied Biosystems).

### Amplification efficiency assay for quantitative real-time RT-PCR primers

A 5-log dilution range was prepared using 10-fold serial dilutions of *N* *. benthamiana* cDNAs. A standard curve based on real-time PCR amplification of this 10-fold serial dilution of template was produced for each pair of real-time primers, by plotting the dilution factor against the C_T_ value obtained for each dilution. All reactions were carried out in triplicate. The equation of the linear regression line, along with Pearson’s correlation coefficient (r), was used and amplification efficiencies (AEs) were calculated using the slope of the regression line: AE = (10^(-1/slope)^ -1) x 100%. All the primer efficiencies are shown in Figure S1. In addition, our quantitative real-time RT-PCR parameters conformed to the minimum information for publication of quantitative real-time PCR experiment (MIQE) guidelines [64]. For comparison of viral RNA levels, Student’s *t*-test was used to calculate significant differences at the 0.05 (*) and 0.01 (**) levels of confidence, respectively. Amplification efficiencies of primer pairs among viral genes and *NbACTIN* (JQ256516) were similar. Similarly, amplification efficiencies of primer pairs among plant host genes and *NbEF1α* (AY206004) were similar. Therefore, *NbACTIN* or *NbEF1α* was used as an internal control for determining viral accumulation or transcriptional level of plant host genes.

### Verification of microarray data and time course study of selected genes

Quantitative real-time RT-PCR was performed to determine viral RNA levels and to investigate the expression of selected host genes of interest, using KAPA SYBR® FAST universal qPCR kit and CFX384^TM^ Real time PCR Detection system (Bio-Rad). The C_T_ values obtained were automatically manipulated by the system. Housekeeping genes *NbACTIN* and *NbEF1α* were chosen as internal controls in the calculation of relative transcript levels. The quantitative real-time RT-PCR was performed as described previously [65].

### Rapid amplification of cDNA ends (RACE) for the open reading frame (ORF) of *NbTOM1*

The ORF of *NbTOM1* was obtained using GeneRacer^®^ Kit with SuperScript^®^ III RT and TOPO TA Cloning^®^ Kit for Sequencing (Invitrogen). The partial sequence of *NbTOM1* (AM261863.1) was used for gene specific primers design. Primers NbTOM1R583 and NbTOM1F347 were used for 5’- and 3’-RACE, following the protocol provided. The resultant PCR products were sequenced using Applied Biosystems 3130 Genetic Analyzer (Applied Biosystems). Amino acid alignment of different homologues of *TOM1* was performed using online software MAFFT version 7 (http://mafft.cbrc.jp/alignment/server/index.html). The alignment result showed that *NbTOM1* is highly conserved. Hydopathy plot of *NbTOM1* was analyzed using online software kyte doolittle hydrophathy plot (http://gcat.davidson.edu/DGPB/kd/kyte-doolittle.htm). Similar to *AtTOM1*, *NbTOM1*also has 7 putative transmembrane regions (Figure S2). The sequence of *NbTOM1* has been uploaded into NCBI database and its accession number is KF051949.

### Construction of vectors for overexpression and silencing of *NbTOM1*

The ORF of *NbTOM1* with restriction enzyme sites PstI and SpeI was amplified by RT-PCR using primers NbTOM1ORFF1PstI, NbTOM1ORFRSpeI and cDNA from young healthy *N* *. benthamiana* . Construct pGreen-*NbTOM1* was obtained by insertion of *NbTOM1*ORF between restriction enzyme sites PstI and SpeI before GFP in pGreen vector. Fragment *NbTOM1*(nt 1-581) with restriction enzyme sites BamHI and XbaI was amplified by RT-PCR using primers NbTOM1F1 BamHI, NbTOMR581 XbaI and cDNA template from young healthy *N* *. benthamiana* . Construct pGreen-*NbTOM1*(nt1-581) was obtained by replacing of GFR fragment in pGreen with *NbTOM1*(nt1-581) between restriction enzyme sites BamHI and XbaI. Constructs pGreen-*NbTOM1*(nt1-581) and pGreen-*NbTOM1* were transformed into *Agrobacterium tumefaciens* GV3101 for overexpression and silencing experiments.

### Agroinfiltration

*Agrobacterium tumefaciens* GV3101 harboring different constructs was subcultured individually in Luria-Bertani (LB) medium with antibiotics (final concentration of Kanamycin 50 µg/ml, Rifampcin50 µg/ml and Tetracycline 5 µg/ml) at 28 °C with shaking until the OD_600_ reading reached 1.0. The bacterial culture was resuspended in Agroinfiltration solution (10 mM MgCl_2_, 0.5 mM acetosyringone and 10 mM 2-(N-morpholino) ethanesulfonic acid (MES) and incubated at RT for 3 h. For the overexpression experiment, 3 leaves per plant (6-8 leaves stage of *N* *. benthamiana* under conditions described earlier under section ‘Plant materials and virus inoculation’, were infiltrated with *A. tumefaciens* GV3101 containing pGreen-*NbTOM1* or pGreen empty vector alone (negative control). Infiltrated leaves at 3 dpa were inoculated with buffer, HLSV or TMV purified virus, following the method and amount described above. Three plants were used for each inoculation. For the silencing experiment, the same types of *N* *. benthamiana* plants were used but infiltrated with *A. tumefaciens* GV3101 containing pGreen-*NbTOM1*(nt1-581) or pGreen alone (negative control). Five days post Agro-infiltration, the same inoculation procedures as described in the overexpression experiment were used. For both overexpression and silencing experiments, total RNA was extracted from virus inoculated *N* *. benthamiana* at 40 h post inoculation (hpi).

## Supporting Information

Figure S1 **Amplification efficiencies of real-time primers.** The amplification efficiencies of primers to be used in quantitative real-time PCR were assayed and found to fall within the 90% to 110% range. Red data points represent candidate primer pairs for the internal control. Blue data points represent primer pairs for selected genes of interest.
(TIF)

Figure S2 **Amino acid alignment of *TOM1* homologues and Hydropathy plot analysis of *NbTOM1*.** (A) Amino acid alignment of different homologues of *TOM1*. (B) Hydopathy plot analysis of *NbTOM1*.
(TIF)

Table S1 **List of primers for reverse transcription, real time RT-PCR, determination of partial sequence, RACE or construction of clones.**
(XLSX)
