# Supplementary figures and images for: Profiling of Genes Related to Cross Protection and Competition for NbTOM1 by HLSV and TMV
Source: PLoS One. 2013 Sep 4;8(9):e73725. doi: 10.1371/journal.pone.0073725 (PMC3762752; doi:10.1371/journal.pone.0073725)

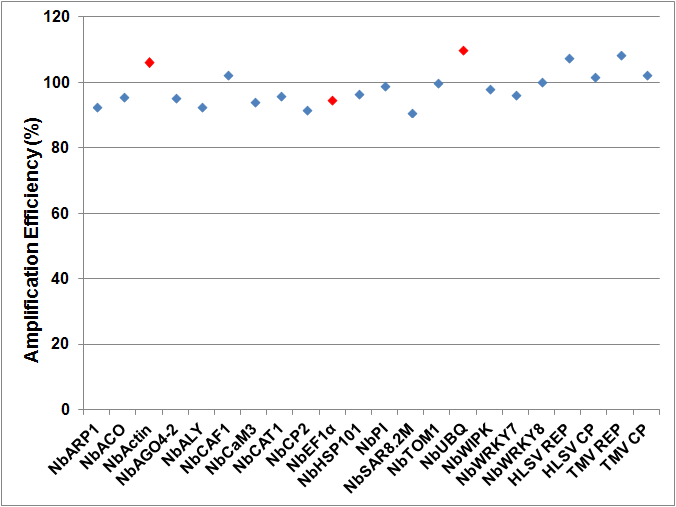

Supplement: Figure S1 — Amplification efficiencies of real-time primers. The amplification efficiencies of primers to be used in quantitative real-time PCR were assayed and found to fall within the 90% to 110% range. Red data points represent candidate primer pairs for the internal control. Blue data points represent primer pairs for selected genes of interest. (TIF) [file pone.0073725.s001.tif]

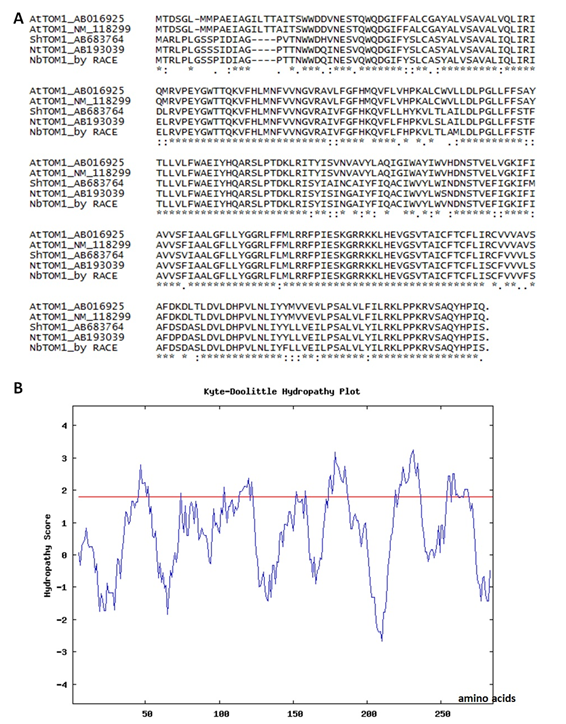

Supplement: Figure S2 — Amino acid alignment of TOM1 homologues and Hydropathy plot analysis of NbTOM1. (A) Amino acid alignment of different homologues of TOM1. (B) Hydopathy plot analysis of NbTOM1. (TIF) [file pone.0073725.s002.tif]
